# Supplementary material for: Systems analysis of miR-199a/b-5p and multiple miR-199a/b-5p targets during chondrogenesis
Source: eLife. 2024 Oct 14;12:RP89701. doi: 10.7554/eLife.89701 (PMC11473111; doi:10.7554/eLife.89701)
Supplement: Supplementary file 1. — (a) Frequency of significant pathways enriched across the chondrogenesis time course. (b) 100 most significantly differentially expressed genes from differential expression analysis which contested day 0 and day 1 chondrogenesis/non-chondrogenesis samples. (c) From the time-course chondrogenesis dataset, we view the log2fc values from 21 predicted 199a/b targets. (d) From two independent chondrogenesis datasets, we view the log2fc values from 21 predicted 199a/b targets. (e) Primers and probes used for the knockdown experiments. (f) Model details for the initial model, including species and ODEs. (g) Model details for the enhanced model, included species, and ODEs. [file elife-89701-supp1.docx]

**Systems analysis of miR-199a/b-5p and multiple miR-199a/b-5p targets during chondrogenesis. Supplementary Materials.**

| ­Wikipathways | D1 | D2 | D3 | D10 | D14 |
| --- | --- | --- | --- | --- | --- |
| Adipogenesis | X | X | X |  |  |
| Apoptosis-related network due to altered Notch3 in ovarian cancer |  |  |  |  | X |
| Burn wound healing |  | X |  |  |  |
| Clear cell renal cell carcinoma pathways |  | X | X | X |  |
| EGF/ EGFR signaling pathway |  | X | X | X | X |
| Endochondral ossification |  | X | X | X | X |
| Endochondral ossification with skeletal dysplasias | X | X | X | X | X |
| Focal adhesion |  |  |  | X | X |
| Focal adhesion: PI3K-Akt-mTOR-signaling pathway |  |  |  | X |  |
| Gastrin Signaling pathway | X | X |  |  | X |
| Hepatitis C and hepatocellular carcinoma |  |  | X |  |  |
| Malignant pleural mesothelioma |  |  |  | X |  |
| Mir-509-3p alteration of YAP1/ ECM axis |  |  | X |  |  |
| Metabolic reprogramming in colon cancer | X | X |  | X |  |
| Oscantin M signaling pathway |  |  |  |  | X |
| PDGF pathway |  |  |  |  | X |
| Retinoblastoma gene in cancer |  |  | X |  |  |
| Spinal cord injury | X |  |  |  |  |
| TGF-beta receptor signaling | X |  |  |  |  |
| TGF-beta receptor signaling in skeletal dysplasias | X |  |  |  |  |
| TGF-beta receptor signaling pathway | X |  |  |  | X |
| Type I collagen synthesis in the context of osteogenesis imperfecta |  | X | X |  |  |
| VEGFA-VEGFR2 signaling pathway | X | X | X | X | X |

**Supplementary file 1a.** The most frequent significantly enriched signalling pathways for each time point based differential expression analyses conducted (DX/ D0) (D=day, D1, D3, D7, D10, D14) are presented. An X marks if the mechanistic pathway was significantly enriched (Benjamini-Hochberg adjusted P value < 0.05) at a that time point.

| log2FoldChange | Padj | name |
| --- | --- | --- |
| 2.616518 | 0 | TGFBI |
| 2.709744 | 0 | TNC |
| 8.394896 | 0 | IGFBP5 |
| 3.538533 | 0 | DCN |
| 1.611538 | 0 | PGK1 |
| 5.15687 | 0 | ANGPTL4 |
| 2.829109 | 0 | IL1R1 |
| 5.613852 | 0 | TSC22D3 |
| -1.88916 | 0 | TUBA1B |
| 3.388879 | 0 | PLIN2 |
| -2.54946 | 0 | NRP1 |
| 2.354566 | 0 | P4HA2 |
| 2.77377 | 0 | COL7A1 |
| 2.607228 | 0 | UGP2 |
| 2.625552 | 0 | DUSP1 |
| 6.370805 | 0 | FKBP5 |
| 3.795323 | 0 | PPP1R3C |
| 2.315841 | 0 | BNIP3 |
| 3.197938 | 0 | SNAI2 |
| 5.970173 | 0 | MT1X |
| 2.234107 | 0 | SLC2A1 |
| 2.184884 | 0 | STEAP3 |
| -2.30296 | 0 | SHISAL1 |
| 6.928209 | 0 | COMP |
| 5.666913 | 0 | HILPDA |
| 3.367955 | 0 | APOL2 |
| 3.824101 | 0 | CRYAB |
| 5.081568 | 0 | PRG4 |
| 3.115528 | 0 | MXI1 |
| 2.436917 | 0 | FOXO3 |
| -2.85381 | 0 | COLEC12 |
| 5.293173 | 0 | BMP2 |
| -3.16176 | 0 | EXT1 |
| 3.374564 | 0 | SOX9 |
| 4.771362 | 0 | ARHGEF19 |
| 2.938684 | 0 | FAM162A |
| -3.75609 | 0 | ADAMTS5 |
| 4.84389 | 0 | FOXO1 |
| -4.97391 | 0 | CLDN11 |
| -3.92651 | 0 | CXCL12 |
| -5.09578 | 7.06E-304 | RGS4 |
| 2.825376 | 9.64E-298 | NMB |
| 2.813253 | 5.26E-297 | SLC6A8 |
| -2.37377 | 2.83E-292 | SH2B3 |
| -1.99395 | 1.87E-288 | HAS2 |
| 2.708725 | 2.49E-287 | PFKFB4 |
| 2.119636 | 1.30E-283 | BGN |
| -1.83236 | 4.99E-277 | ANXA1 |
| 3.210302 | 1.70E-276 | SNAI1 |
| 2.847524 | 4.07E-275 | SAT1 |
| 3.967469 | 5.75E-275 | DEPP1 |
| 4.137285 | 1.10E-274 | SEC14L2 |
| 1.384284 | 4.58E-269 | SH3PXD2A |
| -2.5886 | 3.37E-267 | CDC42EP3 |
| 4.743967 | 1.10E-264 | ATP1B1 |
| -3.81618 | 3.82E-263 | DKK1 |
| -1.97544 | 2.30E-262 | BCL2L1 |
| 1.427985 | 3.96E-262 | ATP1A1 |
| -1.62554 | 1.10E-261 | COTL1 |
| 2.248828 | 2.02E-259 | PYGL |
| 1.666937 | 2.93E-258 | SPARC |
| 6.06187 | 7.90E-258 | ROS1 |
| 2.285569 | 9.94E-258 | KLF9 |
| 3.22922 | 1.16E-245 | TXNIP |
| 3.340382 | 1.65E-242 | DDIT4 |
| -2.96542 | 2.15E-242 | DCBLD2 |
| 1.948309 | 7.83E-242 | COL5A2 |
| 3.433647 | 6.86E-237 | GLUL |
| -2.75208 | 1.75E-235 | FHL2 |
| 2.079477 | 6.83E-235 | COL3A1 |
| 9.995813 | 1.32E-232 | SERPINA3 |
| 2.153327 | 9.18E-227 | TUT7 |
| 3.781249 | 1.48E-226 | COL11A1 |
| 2.563916 | 8.36E-224 | RHOB |
| -1.62741 | 3.09E-219 | CAPN2 |
| 3.888793 | 5.01E-219 | NFIL3 |
| 2.433366 | 3.06E-214 | ZNF395 |
| 2.802341 | 2.29E-213 | SAP30 |
| -2.12046 | 2.45E-212 | NQO1 |
| 3.171023 | 2.98E-205 | PLPP1 |
| 4.079239 | 7.13E-204 | MGP |
| 4.916167 | 4.66E-201 | MMP3 |
| 1.484742 | 2.22E-200 | ATF4 |
| 3.424358 | 3.38E-200 | ACSL1 |
| -1.94314 | 1.12E-198 | GLIPR1 |
| -1.45435 | 5.81E-197 | CFL1 |
| 6.270373 | 3.23E-194 | MAOA |
| 3.667951 | 3.31E-192 | TYRO3 |
| -2.55527 | 1.41E-190 | ADGRL4 |
| -2.3719 | 5.15E-189 | SLC20A1 |
| -2.53549 | 4.76E-188 | SCARA3 |
| 2.350622 | 3.37E-186 | TIPARP |
| 2.674425 | 9.07E-186 | BTG1 |
| 2.042468 | 4.15E-183 | MIOS |
| 3.66087 | 9.66E-182 | NDUFA4L2 |
| 1.49663 | 1.46E-181 | FAM168A |
| -1.11214 | 1.57E-181 | CAV1 |
| -2.5923 | 4.55E-180 | CRIM1 |
| 1.043972 | 8.22E-180 | COL6A3 |
| 3.216222 | 3.72E-178 | PNRC1 |

**Supplementary file 1b.** Log 2-Fold changes, BH adjusted P values and gene symbols from the first 100 most significantly (smallest adjusted P values) genes from performing differential expression analysis to contrast control/ undifferentiated chondrogenesis samples measured at day 1 of chondrogenesis against control/ undifferentiated chondrogenesis samples measured at day 0 of chondrogenesis.

| Gene | Barter  D1/D0 | Barter  D3/D0 | Barter  D6/D0 | Barter  D10/D0 | Barter  D14/D0 |
| --- | --- | --- | --- | --- | --- |
| *ABHD17C* | NA | NA | NA | NA | NA |
| *ATP13A2* | -0.05 | -0.171 | -0.211 | -0.090 | **-0.341** |
| *CAV1* | **-3.380** | **-3.319** | **-1.522** | **-2.013** | **-1.998** |
| *CTSL* | NA | NA | NA | NA | NA |
| *DDR1* | **1.091** | **0.855** | **1.17** | **1.27** | **1.571** |
| *FZD6* | **-1.074** | **-0.781** | **-1.160** | **-1.087** | **-1.074** |
| *GIT1* | -0.007 | 0.071 | -0.156 | -0.118 | -0.044 |
| *HIF1A* | 0.075 | 0.646 | 0.280 | -0.193 | 0.075 |
| *HK2* | 0.141 | 0.401 | 0.111 | -0.051 | 0.141 |
| *HSPA5* | NA | NA | NA | NA | NA |
| *ITGA3* | **-2.289** | **-2.463** | **-2.063** | **-2.689** | **-2.289** |
| *M6PR* | **-0.687** | -0.264 | **-0.871** | -0.285 | **-0.697** |
| *MYH9* | **-1.756** | **-1.742** | **-2.554** | **-2.105** | **-1.756** |
| *NECTIN2* | NA | NA | NA | NA | NA |
| *NINL* | **-0.511** | **0.406** | **-0.364** | **-0.493** | **-0.511** |
| *PDE4D* | **-0.283** | **-0.406** | 0.082 | -0.152 | **-0.283** |
| *PLXND1* | **0.322** | **0.313** | **0.498** | **0.309** | **0.322** |
| *PXN* | -0.786 | -0.228 | -0.148 | -0.172 | -0.168 |
| *SLC35A3* | **0.996** | **0.791** | **1.314** | **1.212** | **0.996** |
| *SLC35D1* | **1.483** | **1.391** | **1.665** | **1.652** | **1.483** |
| *VPS26A* | NA | NA | NA | NA | NA |

**Supplementary file 1c.** Log2fc values from the chondrogenesis microarray study. The 21 genes shown were found through bioinformatic analysis of the RNAseq data. Values that are bold were significantly differentially expressed (adjusted P value is less than 0.05). NA means the gene were not found in the study.

| Gene | Huynh  D1/D0 | Huynh  D3/D1 | Huynh  D7/D3 | Huynh  D14/  D7 | Huynh  D21/  D7 | Huynh  D21/  D14 | Huang  MSC28/  MSC0 | Huang  c28/  MSC0 | Huang  c28/  MSC28 |
| --- | --- | --- | --- | --- | --- | --- | --- | --- | --- |
| *ABHD17C* | **-1.15** | -0.417 | 0.107 | -0.373 | -0.173 | 0.201 | -0.222 | -0.214 | 0.007 |
| *ATP13A2* | -0.387 | -0.698 | **0.577** | -0.06 | -0.208 | -0.14 | -0.07 | 0.355 | 0.425 |
| *CAV1* | **-0.99** | -0.221 | **-1** | -0.323 | 0.0429 | 0.366 | -0.572 | -0.207 | 0.365 |
| *CTSL* | **-0.957** | -0.0991 | -0.518 | -0.279 | -0.177 | 0.102 | NA | NA | NA |
| *DDR1* | 1.18 | -0.681 | -0.145 | 0.076 | 0.2 | 0.123 | 0.395 | **0.636** | 0.241 |
| *FZD6* | -0.362 | **-0.763** | **-0.367** | 0.104 | **0.41** | 0.306 | NA | NA | NA |
| *GIT1* | 0.188 | 0.0364 | **-0.352** | -0.354 | **-0.48** | -0.126 | **-0.472** | -0.354 | 0.238 |
| *HIF1A* | -0.686 | 0.011 | 0.059 | **0.581** | **0.759** | 0.178 | **0.56** | 0.344 | -0.216 |
| *HK2* | 0.556 | -0.212 | **-1.99** | **-0.645** | -0.552 | 0.093 | -0.325 | **0.832** | **1.16** |
| *HSPA5* | **2.89** | -0.443 | **-1.01** | **-1.42** | **-1.51** | -0.089 | NA | NA | NA |
| *ITGA3* | **-1.57** | **-1.01** | -0.26 | -0.729 | -0.019 | 0.709 | **-2.94** | **-4.78** | **-1.84** |
| *M6PR* | -0.269 | 0.056 | -0.005 | 0.0286 | -0.086 | -0.115 | **-0.775** | **-0.991** | -0.216 |
| *MYH9* | -0.236 | **-0.833** | 0.276 | -0.144 | **-0.427** | -0.283 | **-1.69** | **-0.952** | **0.734** |
| *NECTIN2* | NA | NA | NA | NA | NA | NA | NA | NA | NA |
| *NINL* | **-1.18** | **0.513** | 0.176 | -0.153 | 0.133 | -0.02 | NA | NA | NA |
| *PDE4D* | 0.909 | **-1.01** | 0.278 | -0.162 | 0.425 | 0.587 | **0.605** | **0.684** | 0.078 |
| *PLXND1* | **-0.608** | **-0.81** | 0.039 | 0.139 | 0.173 | 0.034 | **-1.46** | **-1.59** | -0.134 |
| *PXN* | **-1.01** | **-0.599** | 0.02 | -0.226 | -0.123 | 0.103 | NA | NA | NA |
| *SLC35A3* | 0.424 | -0.278 | -0.278 | 0.175 | 0.343 | 0.168 | NA | NA | NA |
| *SLC35D1* | 0.381 | -0.143 | 0.616 | 0.764 | **0.798** | 0.0345 | **-1.18** | 0.158 | **1.34** |
| *VPS26A* | -0.949 | -0.364 | -0.278 | -0.397 | -0.114 | 0.283 | -0.773 | -1.19 | -0.422 |

**Supplementary file 1d.** Log2fc values from the 21 *miR-199a/b-5p* targets from bioinformatic analysis of the RNAseq data. Results from *Huynh et al (2019)* and *Huang et al (2010)* are depicted since these are present in the datasets within the *SkeletalVis* repository and identified with the keywords “Chondrogenesis” and “MSC differentiation”. Significantly differentially expressed genes with an adjusted P value of less than 0.05 are bold. NA means the gene was not found in the study. Data from *Huynh et al (2019)* consisted of time points: days 0 1, 3, 7, 14 and 21 after chondrogenesis initiation and the data underwent step-wise DE analysis in *SkeletalVis*. Data from *Huang et al (2010)* consisted of chondrogenesis (c) or MSC samples measured at days 0 or 28, and these have been contrasted in *SkeletalVis*.

| Primer | Sequence |
| --- | --- |
| SOX9 F | ACTTGCACAACGCCGAG |
| SOX9 R | CTGGTACTTGTAATCCGGGTG |
| COL2A1 F | AACCAGATTGAGAGCATCCG |
| COL2A1 R | ACCTTCATGGCGTCCAAG |
| ACAN F | AGCGAGTTGTCATGGTCTG |
| ACAN R | TGTGGGACTGAAGTTCTTGG |
| FZD6 F | GAAGCAAAAAGACATGCACAGA |
| FZD6 R | TTCGACTTTCACTGATTGGATCT |
| ITGA3 F | GAGGACATGTGGCTTGGAGT |
| ITGA3 R | GTAGCGGTGGGCACAGAC |
| CAV1 F | ACAGCCCAGGGAAACCTC |
| CAV1 R | CGGATGGGAACGGTGTAG |
|  |  |
| hFZD6 IFC F | GCTCGCTAGCCTCGATCTCTCGTTACTCAGAAGCAAA |
| hFZD6 IFC R | CGACTCTAGACTCGATGGCACTAATATCGCTATCACAC |
| hITGA3 IFC F | GCTCGCTAGCCTCGACGGACCCGCTATTATCAGATC |
| hITGA3 IFC R | CGACTCTAGACTCGACTGGGAGCTGTTTATTGGTCG |
| hFZD6 mut F | GTGCATAGGTCACTTCGACTCTAACACAAATTTGCTTCTGAGTAACGAGA |
| hFZD6 mut R | TCTCGTTACTCAGAAGCAAATTTGTGTTAGAGTCGAAGTGACCTATGCAC |
| hITGA3 mut1 F | GTGGCTCAAGATGGATCGACTCTGAAAGGGGGAGGTGTC |
| hITGA3 mut1 R | GACACCTCCCCCTTTCAGAGTCGATCCATCTTGAGCCAC |

|  |
| --- |
| \| Probe \|  \|  \|  \|  \| \| --- \| --- \| --- \| --- \| --- \| \| 5'-FAM-TCCTTTGGTCGCTCGCTCCTCTCCC-TAMRA-3' \| \| \| \| \| \|  \|  \|  \|  \|  \| \| 5'-FAM-TCTGGAGACTTCTGAACGAGAGCGA-IABkFQ-3' \| \| \| \| \| \|  \|  \|  \|  \|  \| \| 5'-FAM-AGACCTGAAACTCTGCCACCCTG-IABkFQ-3' \| \| \| \| \| \|  \|  \|  \|  \|  \| \| 5'-FAM-CTGGGTTTTCGTGACTCTGAGGGT-IABkFQ-3' \| \| \| \| \| \|  \|  \|  \|  \|  \| |

| Cloning |  |
| --- | --- |
| hFZD6 IFC F | GCTCGCTAGCCTCGATCTCTCGTTACTCAGAAGCAAA |
| hFZD6 IFC R | CGACTCTAGACTCGATGGCACTAATATCGCTATCACAC |
| hITGA3 IFC F | GCTCGCTAGCCTCGACGGACCCGCTATTATCAGATC |
| hITGA3 IFC R | CGACTCTAGACTCGACTGGGAGCTGTTTATTGGTCG |
| hFZD6 mut F | GTGCATAGGTCACTTCGACTCTAACACAAATTTGCTTCTGAGTAACGAGA |
| hFZD6 mut R | TCTCGTTACTCAGAAGCAAATTTGTGTTAGAGTCGAAGTGACCTATGCAC |
| hITGA3 mut1 F | GTGGCTCAAGATGGATCGACTCTGAAAGGGGGAGGTGTC |
| hITGA3 mut1 R | GACACCTCCCCCTTTCAGAGTCGATCCATCTTGAGCCAC |

**Supplementary file 1e.** Sequences of primers and probes used for mutagenesis experiments and/or knock-downs.

**Supplementary file 1f.** Model Specifics for the initial chondrogenesis model.

**Initial conditions**

| Model species | Value (mmol/ml) | Description |
| --- | --- | --- |
| *Acan* mRNA | 6.257096819 | Chondrogenesis biomarker. |
| *CAV1* mRNA | 13.21139434 | General genetic activity of CAV1 mRNA and protein. |
| *Col2a1* mRNA | 6.40998058 | Chondrogenesis biomarker. |
| *FZD6* mRNA | 8.692679393 | General genetic activity of FZD6 mRNA and protein. |
| GAG | 1 | Chondrogenesis biomarker. |
| HP199a | 0 | Drug reduces miR-199a-5p when event is triggered. |
| HP199b | 0 | Drug reduces miR-199b-5p when event is triggered. |
| *ITGA3* mRNA | 8.925880317 | General genetic activity of ITGA3 mRNA and protein. |
| miR-199a-5p | 10.5401385 | miRNA which modulates FZD6, ITGA3 and CAV1. |
| miR-199b-5p | 5.423464 | miRNA which modulates FZD6, ITGA3 and CAV1. |
| *SOX9* mRNA | 8.76039807 | Chondrogenesis biomarker. |

**Events**

In this kinetic model we have four events (HP199a activity, HP199a inactivity, HP199b activity, HP199b inactivity) have been used to simulate behaviours seen in our *miR-199a-5p* and *miR-199b-5p* knockdown experiments. When triggered, HP199a activity and HP199a inactivity both together lead to HP199a = 1 until time reaches 7 days, at which point HP199a = 0. Likewise, when both HP199b activity and HP199b inactivity are triggered, they lead to lead to HP199b = 1 until time reaches 7 days, at which point HP199a = 0. HP199a and HP199b will reduce their target miRNA by 90%-95% until day 7. HP199a and HP199b modulate global quantities which are fixed at 1. When not triggered the global quantities will not be modulated.

**Ordinary Differential Equations (ODEs)**

This was an ODE based kinetic model. Each of the model species have inputs and outputs which modulate their behaviours over the 14-day time course. All parameters have been rounded up to three decimal places. ch = chondrocyte compartment. ecm = extracellular matrix. ch and ecm were compartments, both equalled 1 so performed no modulation.

$$\frac{d\left[ MIR199b\_5p \right].ch}{dt}=[+ ch . 4.60528$$

$$-ch.(20 . \left[ MIR199b\_5p \right]. [HP199b])$$

$$- ch.(0.505.[MIR199b\_5p]) ]$$

$$\frac{d\left[ MIR199a\_5p \right].ch}{dt}=[+ ch . 104.738$$

$$-ch.(201.443 . \left[ MIR199a\_5p \right]. [HP199a])$$

$$- ch.(9.066 .[MIR199a\_5p]) ]$$

$$\frac{d\left[ ACAN\_mRNA \right].ch}{dt}=[+ ch . (400.198 . [SOX9\_mRNA])$$

$$-ch.(507.529. \left[ ACAN_{mRNA} \right] )]$$

$$\frac{d\left[ COL2A1\_mRNA \right].ch}{dt}=[+ ch . (778.139 . [SOX9\_mRNA])$$

$$-ch.(693.578.529. \left[ COL2A1\_mRNA \right]) ]$$

$$\frac{d\left[ SOX9\_mRNA \right].ch}{dt}=[+ ch . 5000$$

$$-ch.(10 . \left[ SOX9\_mRNA \right] . [FZD6\_mRNA] )$$

$$-ch. \left( \left[ SOX9\_mRNA \right]. \left[ ITGA3\_mRNA \right] \right)$$

$$-ch. \left( \left[ SOX9\_mRNA \right] . [CAV1\_mRNA] \right)]$$

$$\frac{d\left[ FZD6\_mRNA \right].ch}{dt}=[+ ch . (2051.29)$$

$$-ch.\left( 10. \left[ FZD6\_mRNA \right] . [MIR199a\_5p] \right)$$

$$- ch. (18 . [FZD6\_mRNA] . [MIR199b\_5p])]$$

$$\frac{d\left[ ITGA3\_mRNA \right].ch}{dt}=[+ ch . (1732.04)$$

$$-ch.\left( 10. \left[ ITGA3\_mRNA \right] . [MIR199a\_5p] \right)$$

$$- ch. (18 . [ITGA3\_mRNA] . [MIR199b\_5p])]$$

$$\frac{d\left[ CAV1\_mRNA \right].ch}{dt}=[+ ch . (1732.04)$$

$$-ch.\left( 10. \left[ CAV1\_mRNA \right] . [MIR199a\_5p] \right)$$

$$- ch. (18 . [CAV1\_mRNA] . [MIR199b\_5p])]$$

**Supplementary file 1g.** Model specifics for the enhanced chondrogenesis model.

**Initial conditions**

| Model species | Value (mmol/ml) | Description |
| --- | --- | --- |
| *Acan* mRNA | 6.257096819 | Chondrogenesis biomarker. |
| CAV1 | 13.21139434 | General genetic activity of CAV1 mRNA and protein. |
| *Col2a1* mRNA | 6.40998058 | Chondrogenesis biomarker. |
| FZD6 | 8.692679393 | General genetic activity of FZD6 mRNA and protein. |
| GAG | 1 | Chondrogenesis biomarker. |
| HP199a | 0 | Drug reduces *miR-199a-5p* when event is triggered. |
| HP199b | 0 | Drug reduces *miR-199b-5p* when event is triggered. |
| ITGA3 | 8.925880317 | General genetic activity of ITGA3 mRNA and protein. |
| *miR-140-5p* | 6.5582925 | miRNA which modulates FZD6 and biomarker. |
| *miR-199a-5p* | 10.5401385 | miRNA which modulates FZD6, ITGA3 and CAV1. |
| *miR-199b-5p* | 5.423464 | miRNA which modulates FZD6, ITGA3 and CAV1. |
| OtherTargets | 25 | Representative of other *199a/b* targets. |
| OtherTargetsRegulators | 1000 | Regulator of OtherTargets to help modulation. |
| *SOX9* mRNA | 8.76039807 | Chondrogenesis biomarker. |
| SOX9PhosphoProtein | 0 | Promotes *ACAN*, *COL2A1*, *miR-140-5p.* |
| SOX9 | 1 | Promotes SOX9PhosphoProtein. |
| SRC | 1000 | Modulator of CAV1. |
| TGFB3 | 10000 | Represents chondrogenesis initiation. |

**Events**

In this kinetic model we have four events (HP199a activity, HP199a inactivity, HP199b activity, HP199b inactivity) have been used to simulate behaviours seen in our *miR-199a-5p* and *miR-199b-5p* knockdown experiments. When triggered, HP199a activity and HP199a inactivity both together lead to HP199a = 1 until time reaches 4.5 days, at which point HP199a = 0. Likewise, when both HP199b activity and HP199b inactivity are triggered, they lead to lead to HP199b = 1 until time reaches 4.5 days, at which point HP199a = 0. HP199a and HP199b will reduce their target miRNA by 90%-95% until day 4.5. HP199a and HP199b modulate global quantities which are fixed at 1. When not triggered the global quantities will not be modulated.

**Ordinary Differential Equations (ODEs)**

This was an ODE based kinetic model. Each of the model species have inputs and outputs which modulate their behaviours over the 14-day time course. All parameters have been rounded up to three decimal places. ch = chondrocyte compartment. ecm = extracellular matrix. ch and ecm were compartments, both equalled 1 so performed no modulation.

$$\frac{d\left[ ACAN mRNA \right].ch}{dt}=[+ ch.(\frac{100.[Sox9PhosphoProtein]}{1+\frac{1}{[Sox9PhosphoProtein]}}$$

$$- ch.(4.263.[ACAN mRNA]) ]$$

$$\frac{d\left[ CAV1 \right].ch}{dt}=[+ch.\frac{152.229 . 9625.57.[SRC]}{3.\left( 0.416+\left[ SRC \right] \right)+9625.57 . (0.1+[SRC])}$$

$$- ch.\frac{12.892.\left[ CAV 1 \right]}{0.0972+\left[ CAV 1 \right]+0.0971 .\frac{[miR-199a-5p]}{0.1318}} . [miR-199a-5p]$$

$$- ch.\frac{18.746.\left[ CAV 1 \right]}{0.0996+\left[ CAV 1 \right]+0.0996 .\frac{[miR-199b-5p]}{0.057}} . [miR-199b-5p]$$

$$- ch.(0.267.[CAV1]) ]$$

$$\frac{d\left[ COL2A1 mRNA \right].ch}{dt}=[+ch. (\frac{94.624 . [Sox9PhosphoProtein]}{1+\frac{1}{[Sox9PhosphoProtein]}})$$

$$-ch . (3.005 . \left[ COL2A1 mRNA \right]]$$

$$\frac{d\left[ GAG \right].ecm}{dt}=[+ch.( \frac{0.179.\left[ COL2A1 mRNA \right]}{1+89.324}/[COL2A1 mRNA])$$

$$+ch. (\frac{4.70977.\left[ ACAN mRNA \right]}{1+1}/[ACAN mRNA])$$

$$+ch/(\frac{3.97.\left[ miR-140-5p \right]}{1+5}/[miR-140-5pmRNA])]$$

$$\frac{d\left[ miR-140-5p \right].ch}{dt}=[+ch.(6.085.[Sox9PhosphoPrortein])$$

$$-ch.\left( 0.376.\left[ miR-140-5p \right] \right)]$$

$$\frac{d\left[ miR-199a-5p \right].ch}{dt}=[+ch.\frac{100.41. \frac{\left[ TGFB3 \right]}{104.984}}{1+\frac{\left[ TGFB3 \right]}{104.984}/]}$$

$$-ch.(8.678.[miR-199a-5p])$$

$$-ch.\left( \frac{( 1.009.[miR-199a-5p]}{0.017} \right). [HP199a]]$$

$$\frac{d\left[ miR-199b-5p \right].ch}{dt}=[+ch.\frac{8.013 . \frac{\left[ TGFB3 \right]}{0.015}}{1+\frac{\left[ TGFB3 \right]}{0.015}/]}$$

$$-ch.(0.90175.[miR-199b-5p])$$

$$-ch.\left( \frac{( 1.004.[miR-199b-5p]}{0.124} \right). [HP199b]]$$

$$(d[OtherTargets].ch)/dt=[+ch.\frac{1354.23 . 1554.29.\left[ \mathrm{OtherTargetsRegulator} \right]}{0.008 . \left( 0.197+\left[ \mathrm{OtherTargetsRegulator} \right] \right)+1553.9 . \left( 0.009+\left[ \mathrm{OtherTargetsRegulator} \right] \right)})$$

$$-ch .\left( \frac{100.728 . \left[ \mathrm{OtherTargets} \right]}{0.1+\left[ \mathrm{OtherTargets} \right]+0.1\frac{\left[ miR-199a-5p \right]}{0.103}} \right)$$

$$-ch .(\frac{121.391 . [OtherTargets]}{0.095+\left[ \mathrm{OtherTargets} \right]+0.095\frac{[miR-199b-5p]}{0.105}})$$

$$-ch.(0.097.[OtherTargets])]$$

$$\frac{d\left[ OtherTargetsRegulator \right].ch}{dt}=[+ch . 0.099$$

$$-ch. (8.652.\left[ OtherT argetsRegulators \right])]$$

$$\frac{d\left[ SRC \right]. ch}{dt}=[ +ch.(\frac{\left[ TGFB \right]. 0.117}{100})$$

$$-ch. 1.407 . [SRC]]$$

$$\frac{d[SOX9 mRNA]}{dt}\boldsymbol{=[}+ch. (604.499 . \left[ SOX9 mRNA \right])$$

$$+ch.\boldsymbol{(}2.1577.[CAV1]\boldsymbol{)}$$

$$+ch.\boldsymbol{(}1.551.[TGFB3]\boldsymbol{)}$$

$$+ch.\boldsymbol{(}8.44.[miR-140-5p]\boldsymbol{)}$$

$$-ch.\boldsymbol{(}([SOX9 mRNA].11.921.[OtherTargets]\boldsymbol{)}$$

$$-ch.([SOX9mRNA].59.112.[CAV 1])]$$

$$\frac{d[SOX9]}{dt}\boldsymbol{=[}+ch. (604.499 . \left[ SOX9 mRNA \right])$$

$$-ch.(0.376.[SOX9])]$$

$$\frac{d[SOX9PhosphoProtein]}{dt}\boldsymbol{=[}+ch. (1.848)$$

$$-ch.(0.00475.[TGFB3])]$$

$$\frac{d[TGFB3]}{dt}\boldsymbol{=[}+ch. (6.085.[SOX9])$$

$$-ch.(0.376.[0.376.[TGFB3]]]$$
